# Different effects of cortisol on pro-inflammatory gene expressions in LPS-, heat-killed *E.coli*-, or live *E.coli*-stimulated bovine endometrial epithelial cells

**DOI:** 10.1186/s12917-020-2231-z

**Published:** 2020-01-09

**Authors:** Luying Cui, Yali Wang, Heng Wang, Junsheng Dong, Zixiang Li, Jun Li, Chen Qian, Jianji Li

**Affiliations:** 1 College of Veterinary Medicine, Yangzhou University, 48 East Wenhui Rd, Yangzhou, 225009 Jiangsu China; 2 Jiangsu Co-innovation Center for the Prevention and Control of Important Animal Infectious Disease and Zoonoses, 48 East Wenhui Rd, Yangzhou, 225009 Jiangsu China

**Keywords:** Endometritis, Bovine endometrial epithelial cells, Cortisol, *Escherichia coli*, Pro-inflammatory genes

## Abstract

**Background:**

Bacterial infections are common in postpartum dairy cows. Cortisol level has been observed to increase in dairy cows during peripartum period, and is associated with the endometrial innate immunity against pathogens like *E.coli*. However, the mechanism underlying how cortisol regulates *E.coli*-induced inflammatory response in bovine endometrial epithelial cells (BEEC) remains elusive.

**Results:**

Cortisol decreased the expressions of IL1β, IL6, TNF-α, IL8, and TLR4 mRNA in BEEC treated with LPS or heat-killed *E.coli*, but up-regulated these gene expressions in BEEC stimulated by live *E.coli*.

**Conclusion:**

Cortisol exerted the anti-inflammatory action on LPS- or heat-killed *E.coli*-stimulated BEEC, but the pro-inflammatory action on live *E.coli*-induced BEEC.

## Background

Endometritis is the inflammation of the endometrium which usually occurs in postpartum dairy cows. There are two main types of bovine endometritis, including clinical endometritis with purulent (> 50% pus) discharge in uterine lumen between 20 and 33 days postpartum or mucopurulent (approximately 50% pus and 50% mucus) discharge after 26 to 33 days postpartum [1, 2], and subclinical (cytological) endometritis, characterized by the abnormal proportion of polymorphonuclear (PMN) cells in endometrial cytology examination [1]. Endometritis causes infertility at the time the uterine infection is present and subfertility even after successful resolution of the disease, resulting in increased culling rate and great economic loss [2]. The most common cause of uterine infection is the pathogenic microorganisms affecting productivity and fertility of cows. Infection of the endometrium with *Escherichia coli* (*E.coli*) precedes infection by *Trueperella pyogenes* and a range of anaerobic bacteria that include *Fusobacterium, Prevotella* and *Bacteriodes* species [3, 4].

Uterine defenses rely initially on classical innate immunity. The bovine endometrial epithelial cells (BEEC) are the first to make contact with potential pathogens that enter the uterus [5]. One of the critical mechanisms recognizing pathogens and their pathogen associated molecular patterns is Toll-like receptors [6]. It is the Toll-like receptor 4 (TLR4) that specifically recognizes the lipopolysaccharide (LPS), a structural component of the outer membrane of *E.coli* [7, 8]. The activation of TLR4 causes the recruitment of the signaling adaptor MyD88, resulting in the activation of the downstream NF-κB signaling pathways. These signaling cascades stimulate the expressions of pro-inflammatory mRNA transcripts in the endometrium, including the cytokines interleukin1β (IL1β), IL6, tumor necrosis factor-α (TNF-α) and the chemokine IL8 [9, 10].

As a kind of glucocorticoids, cortisol is widely considered as an anti-inflammatory steroid hormone [11–13], which regulates all aspects of immune functions and inflammation [14]. The effects of glucocorticoids are described as inhibiting nuclear translocations and the functions of several pro-inflammatory transcription factors, then suppressing the syntheses of inflammatory mediators [15]. Increased level of endogenic cortisol was observed during parturition [16]. Previous study indicated that cortisol inhibited the inflammatory response in LPS-induced BEEC [17]. However, the relationship between cortisol and the endometritis caused by *E.coli* has not yet been clarified. The effect of cortisol on *E.coli-*stimulated BEEC is worthy of being investigated.

Here we reported the different effects of cortisol on the gene expressions of pro-inflammatory cytokines induced by *E.coli* or LPS. Quantitative PCR method was used to detect the expressions of IL1β, IL6, TNF-α, IL8, and TLR4 mRNA in BEEC co-treated with cortisol and LPS, heat-killed *E.coli* or live *E.coli*.

## Results

### Cell viability

**Fig. 1:**
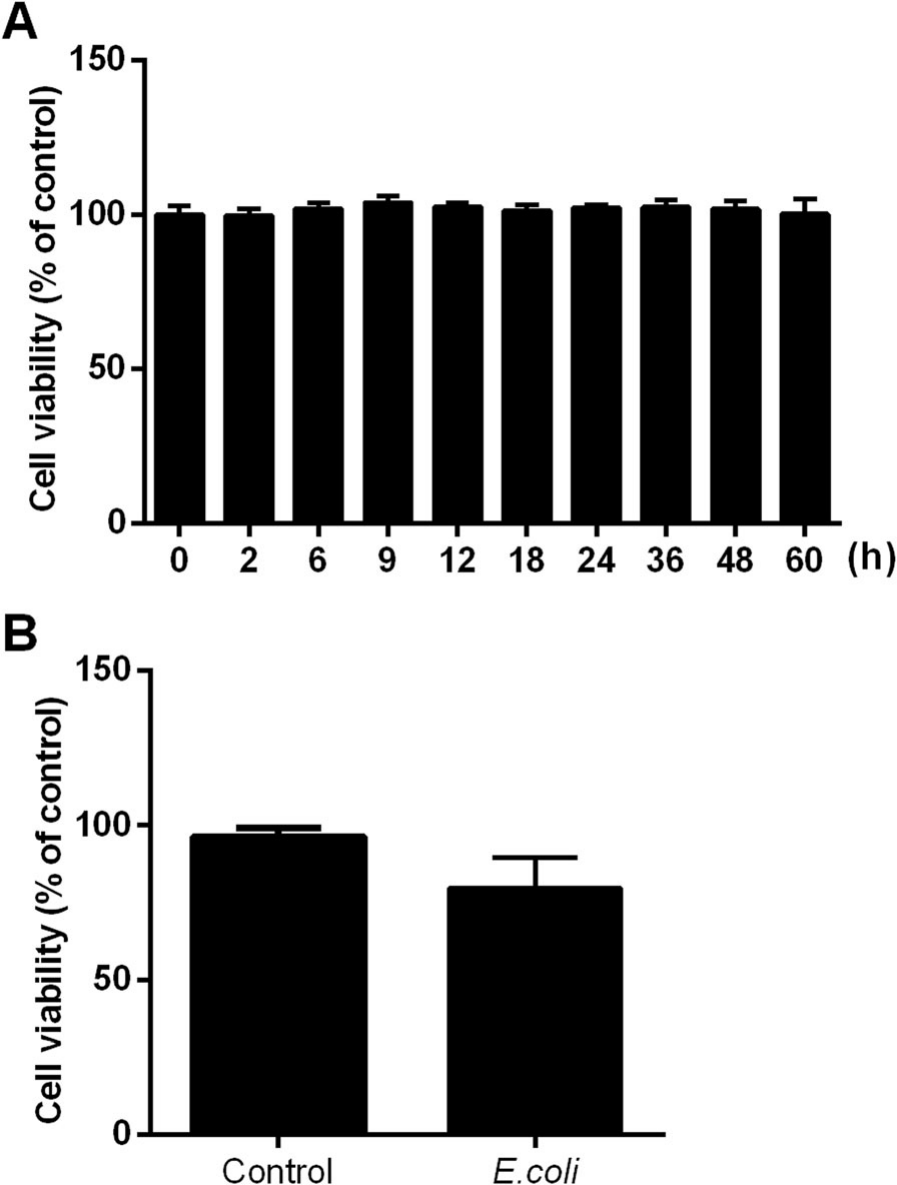
The effect of heat-killed and live *E.coli* on bovine endometrial epithelium cell viability. **a** The cell viabilities of BEEC challenged by 1 × 10^8^ CFU/mL heat-killed *E.coli* from 0 to 60 h were measured by CCK-8 assay. The data were presented as means ± SEM (*n* = 6). **b** The cell viabilities of BEEC challenged by 1 × 10^6^ CFU/mL live *E.coli* at 6 h were measured by trypan blue assay. The data were presented as means ± SEM (*n* = 3)

Cell Counting Kit-8 (CCK-8) and trypan blue assay were used to evaluate the viability of BEEC after heat-killed or live *E.coli* challenge. As shown in Fig. 1a, no difference was observed in cell viability in BEEC treated with heat-killed *E.coli* (1 × 10^8^ CFU/mL) from 0 to 60 h. Compared to the control group, the cell viability was not influenced by live *E.coli* (1 × 10^6^ CFU/mL) at 6 h (Fig. 1b).

### Pro-inflammatory genes and TLR4 mRNA expressions in BEEC co-treated with LPS and cortisol

**Fig. 2:**
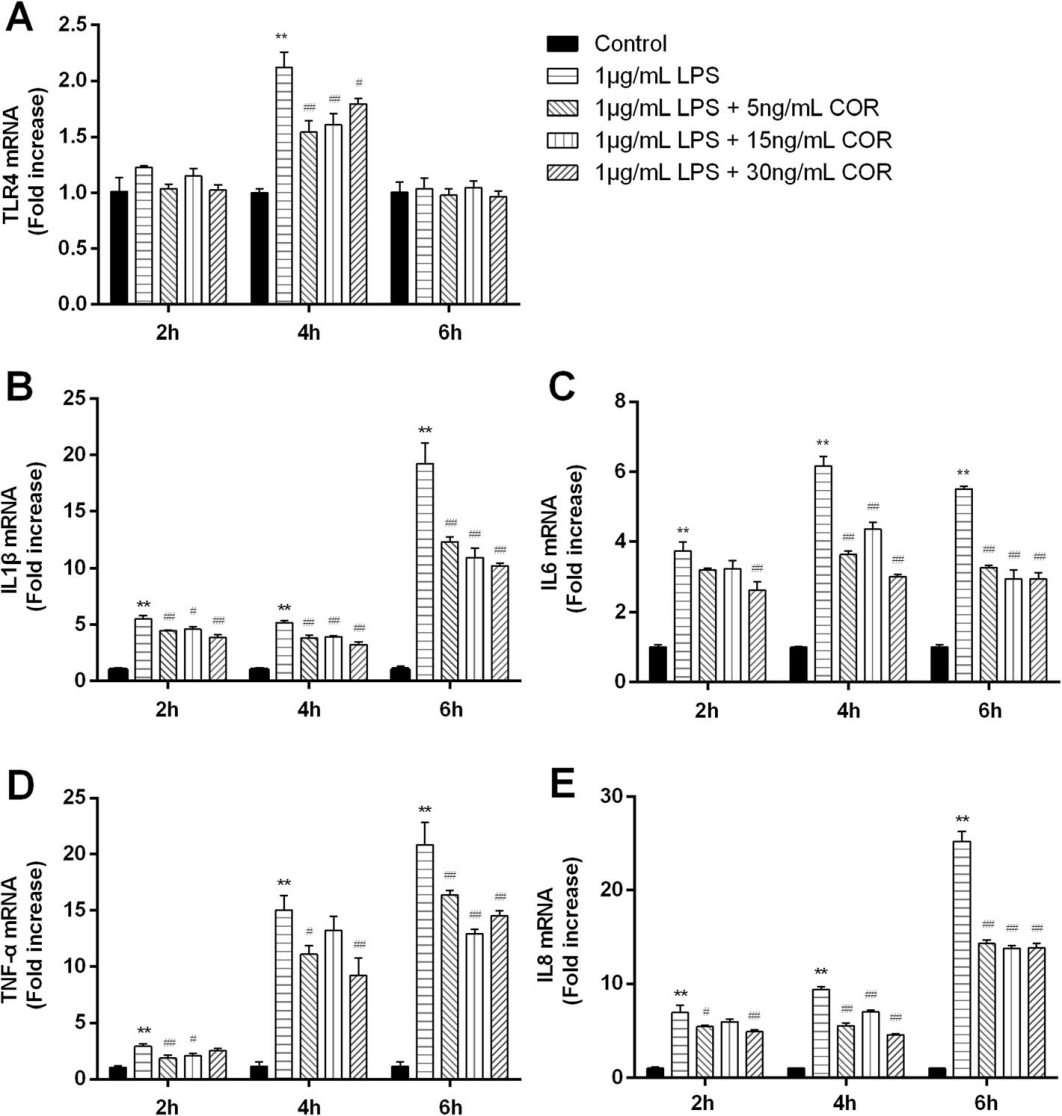
Effects of cortisol on the mRNA expressions of TLR4 (**a**), IL1β (**b**), IL6 (**c**), TNF-α (**d**) and IL8 (**e**) in LPS-stimulated bovine endometrial epithelial cells. Cells were co-treated with cortisol (5, 15, or 30 ng/ mL) and LPS (1 μg/mL) for 2, 6, or 18 h. RNA was isolated and analysed by qPCR. The data were present as means ± SEM (*n* = 3). COR, cortisol. ^**^ *p* < 0.01 vs the control group; ^#^ *p* < 0.05, ^##^ *p* < 0.01 vs the LPS group

The effect of cortisol on the pro-inflammatory and TLR4 mRNA expressions in BEEC stimulated with LPS were shown in Fig. 2. Higher (*p* < 0.01) levels of LPS-induced IL1β, IL6, TNF-α, and IL8 mRNA expressions were observed as compared with the control group at all time points, whereas the TLR4 mRNA increased (*p* < 0.01) only at 6 h after challenge. Compared with *E.coli* treated group, cortisol (5, 15, or 30 ng/mL) decreased (*p* < 0.01) the mRNA expressions of TLR4 at 6 h, and the IL1β, IL6, TNF-α, and IL8 at most observed time points.

### Pro-inflammatory genes and TLR4 mRNA expressions in BEEC co-treated with heat-killed *E.coli* and cortisol

**Fig. 3:**
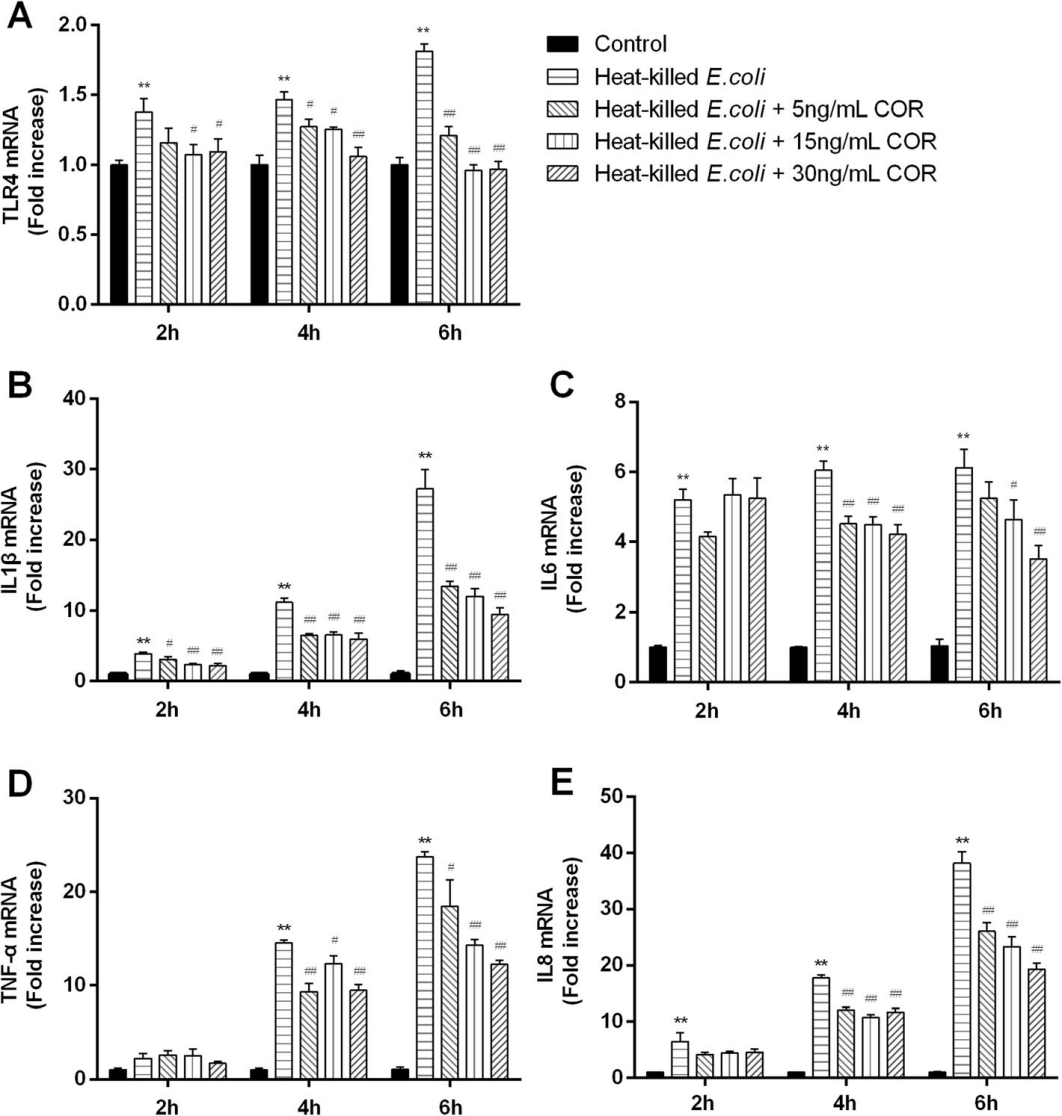
Effects of cortisol on the mRNA expressions of TLR4 (**a**), IL1β (**b**), IL6 (**c**), TNF-α (**d**) and IL8 (**e**) in heat-killed *E.coli*-stimulated bovine endometrial epithelial cells. Cells were co-treated with cortisol (5, 15, or 30 ng/mL) and heat-killed *E.coli* (1 × 10^8^ CFU/mL) for 2, 6, or 18 h. RNA was isolated and analysed by qPCR. The data were present as means ± SEM (*n* = 3). COR, cortisol. ^**^ *p* < 0.01 vs the control group; ^#^ *p* < 0.05, ^##^ *p* < 0.01 vs the heat-killed *E.coli* group

The results of IL1β, IL6, TNF-α, IL8, and TLR4 mRNA expressions in BEEC co-treated with heat-killed *E.coli* and cortisol were shown in Fig. 3. Exposure of cells to heat-killed *E.coli* upregulated (*p* < 0.01) the mRNA expressions of IL1β, IL6, IL8, and TLR4 at all indicated time points, and TNF-α at 6 and 18 h. Compared with *E.coli* treated group, cortisol (5, 15, or 30 ng/mL) treatment generally down-regulated (*p* < 0.05) these gene expressions at 6 and 18 h, and the IL1β and TLR4 mRNA at 2 h.

### Pro-inflammatory genes and TLR4 mRNA expressions in BEEC co-treated with live *E.coli* and cortisol

**Fig. 4:**
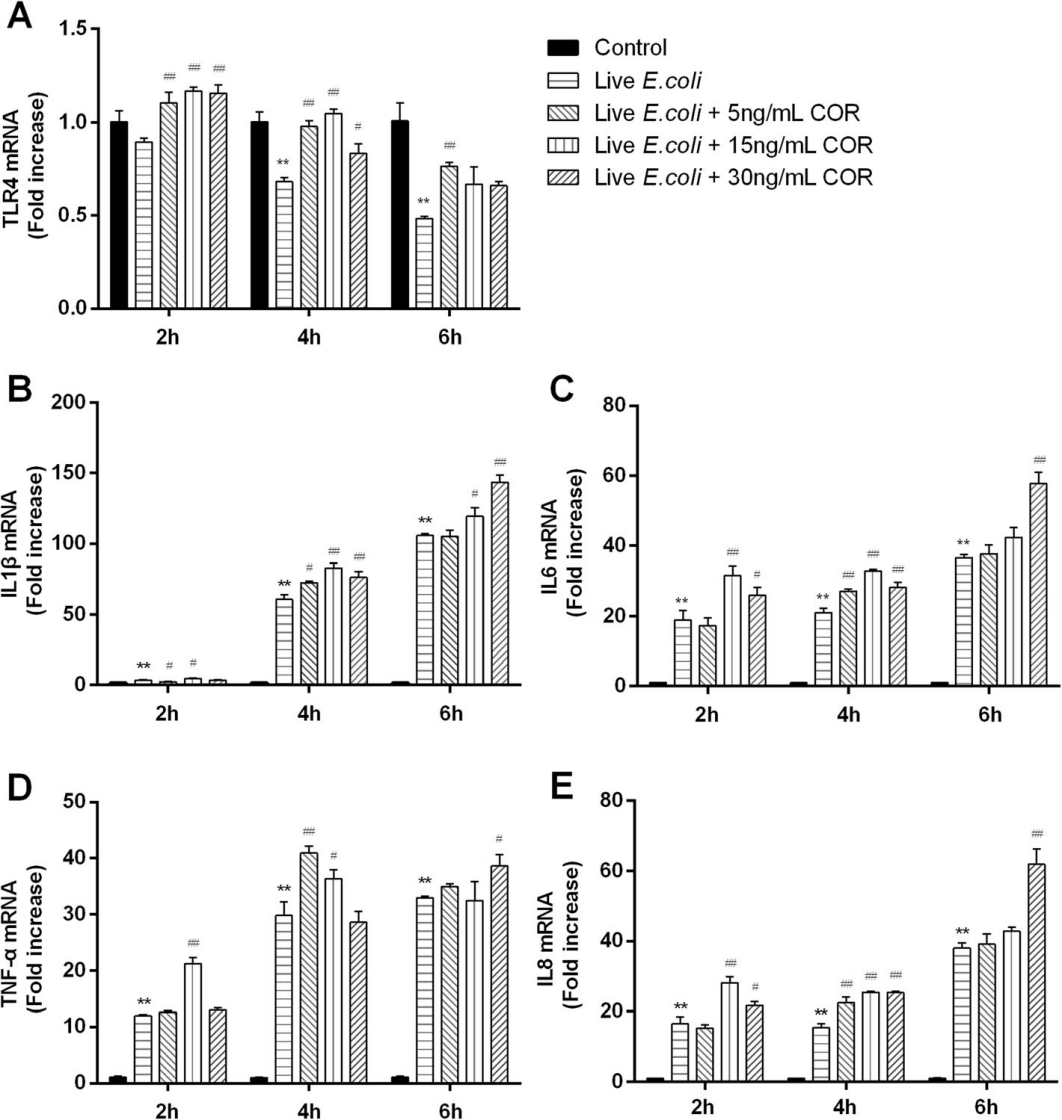
Effects of cortisol on the mRNA expressions of TLR4 (**a**), IL1β (**b**), IL6 (**c**), TNF-α (**d**) and IL8 (**e**) in live *E.coli*-stimulated bovine endometrial epithelial cells. Cells were co-treated with cortisol (5, 15, or 30 ng/mL) and live *E.coli* (1 × 10^6^ CFU/mL) for 2, 4, or 6 h. RNA was isolated and analysed by qPCR. The data were present as means ± SEM (*n* = 3). COR, cortisol. ^**^ *p* < 0.01 vs the control group; ^#^ *p* < 0.05, ^##^ *p* < 0.01 vs the live *E.coli* group

The results of IL1β, IL6, IL8, TNF-α, and TLR4 mRNA expressions were shown in Fig. 4. The mRNA expressions of IL1β, IL6, IL8 and TNF-α increased (*p* < 0.01) by live *E.coli* challenge at all time points. The TLR4 mRNA in *E.coli* group decreased (*p* < 0.01) at 4 and 6 h, and unchanged at 2 h. Compared with *E.coli* treated group, co-treatment of cortisol and *E.coli* generally increased the mRNA expressions of IL1β, IL6, IL8, and TNF-α, which was most pronounced (*p* < 0.05) at 4 h by 5, 15 and 30 ng/mL cortisol, at 2 h by 15 ng/mL cortisol, and at 6 h by 30 ng/mL cortisol. The mRNA levels of TLR4 in the co-treatment group were higher (*p* < 0.05) than the *E.coli* group at 2 and 4 h.

## Discussion

In this study, we demonstrated that cortisol inhibited the LPS- and heat-killed *E.coli-*induced expressions of pro-inflammatory genes (IL-1β, IL-6, IL-8, TNF-α, and TLR4) in BEEC. However, in cells challenged by live *E.coli*, we detected the reduced TLR4 mRNA expression and increased mRNA levels of IL-1β, IL-6, IL-8, and TNF-α. Co-treatment of cortisol and live *E.coli* further up-regulated the expressions of these pro-inflammatory genes.

Increased mRNA expressions of TLR4 have been reported in LPS-stimulated BEEC by Shen et al. [18], Herath et al. [9] and Dong et al. [17], and in heat-killed *E.coli*-stimulated BEEC by Chapwanya et al. [19]. Similarly, Yang et al. showed that heat-killed *E.coli* activated TLR4 receptor in HEK293 cells [20].. This concurs with our study, that LPS and heat-killed *E.coli* could up-regulate TLR4 mRNA. Binding of LPS to TLR4 increases the secretion of inflammatory cytokines [21, 22]. IL1β and IL6 are classical pro-inflammatory cytokines. The activities of IL1β and IL6 are similar to TNF-α, including the induction of pyrexia and the production of acute phase proteins [23]. IL8 is a chemokine that recruits immune cells such as neutrophils and lymphocytes, and stimulates the release of neutrophil granules [24]. Brauner et al. demonstrated that heat-killed *E.coli* induced the protein productions of IL-1β, IL-6, IL-8, and TNF-α [25]. Similarly in our result, the gene expressions of IL-1β, IL-6, IL-8, and TNF-α increased following the stimulation with LPS or heat-killed *E.coli*.

In contrast with increased TLR4 mRNA expression induced by LPS or heat-killed *E.coli*, the live *E.coli* down-regulated TLR4 expression. However, the levels of IL-1β, IL-6, IL-8, and TNF-α mRNA increased after the live *E.coli* stimulation. Previous study indicated that the pathogenic mechanism of *E.coli* is complex. Some pathotypes of *E.coli* have a large repertoire of effectors that are translocated into host cells by the type III secretion system (T3SS) and affect host cell activities [26, 27]. T3SS can induce an early pro-inflammatory response through the activation of NF-κB [28], resulting in the increased expression of pro-inflammatory genes. Another possibility was that several pathogenic Gram-negative bacteria have a modified lipid A, which is a part of LPS and enables the bacteria to evade TLR4-mediated immune surveillance [29]. This possibility was less likely because of the increased TLR4 expression in heat-killed *E.coli* group. Therefore, we speculated that the underlying mechanism of the increased expressions of inflammatory genes was not mediated by TLR4. We were unable to explain why the TLR4 gene expression decreased after the stimulation of live *E.coli*, which may require further investigation on TLR4 at posttranscriptional level and on the interaction between TLR4 and live *E.coli*.

It has been accepted that glucocorticoids induce anti-inflammatory effects by inhibiting the expressions of many pro-inflammatory genes [30] and the signal transduction of pattern recognition receptors [31]. According to the previous studies by our lab, cortisol inhibited the LPS-induced mRNA expressions of TLR4, IL-1β, IL-6, IL-8, and TNF-α in BEEC [17]. In agreement with these reports, we found that cortisol exerted the classical anti-inflammatory effects on BEEC induced with LPS or heat-killed *E.coli.* However, cortisol increased the levels of IL-1β, IL-6, IL-8, TNF-α, and TLR4 mRNA in BEEC treated with live *E.coli*, exacerbating the pro-inflammatory effects. It has been reported that glucocorticoids have not only universally anti-inflammatory actions, but also pro-inflammatory effects in acute stress situations [32, 33]. The pro-inflammatory effects including increased expressions of chemokines, complement proteins, and cytokines [33], and inducing the secretion and activation of TLR signaling pathway [31, 32]. We hypothesized that the live *E.coli* stimulation may cause acute stress of cells, resulting in the pro-inflammatory effects of cortisol. There is a substantial space for further research to determine the condition and underlying mechanism of how the pro-inflammatory effects occur.

## Conclusion

The present study demonstrated a two-side effect of cortisol in BEEC stimulated with LPS, heat-killed *E.coli* or live *E.coli*. Cortisol inhibited the LPS- or heat-killed *E.coli-*induced inflammatory response, but up-regulated the live *E.coli-*induced inflammatory response.

## Methods

### Endometrial epithelium cell culture

The cells were isolated as described from Dong et al. [17] and from Cui et al. [34]. Cattle with hoof disease or mastitis from experimental farm of Yangzhou University were sent to the local abattoir and were culled. Bovine uteri from postpubertal nonpregnant cattle with no evidence of genital disease or microbial infection were collected and kept on ice until further processing in the laboratory [10]. Briefly, the uterine horns were cut and digested with 0.1% protease from *Streptomyces griseus* (P5147, Sigma, USA) diluted in DMEM/F-12 (D8900, Sigma, USA) at 4 °C for 18 h. Then the uterine horns were incised to facilitate scraping the endometrium. The scraped materials were washed, collected and centrifuged at 1000×*g* for 5 min. The cells were resuspended in DMEM/F-12 containing 15% fetal bovine serum (FBS, Gibco, USA) and 50 U/mL of penicillin/streptomycin. These cells were seeded in 25 cm^2^ flasks and cultured at 37 °C containing 5% CO_2_. The medium was changed when the cells reached 90% confluence. The purification of BEEC was confirmed to be above 99% by detecting CK-18 using immunohistochemistry.

### Bacteria

*E.coli* O55 was obtained and isolated from a cow with endometritis (purulent uterine discharge) at experimental farm of Yangzhou University. The strain was routinely cultured overnight (37 °C, 120 rpm) in nutrient LB broth (L3022, Sigma, USA), and the bacterial fluid was pipetted into sterile LB for incubating (37 °C, 120 rpm) until to an optical density (OD 600 nm) of 0.6 to have a final density to 4 × 10^8^ ± 1 × 10^8^ CFU/mL. Both active and heat-inactive *E.coli* were used in this study. For live *E.coli*, the bacterial suspension was centrifuged at 4000×*g* for 10 min and followed twice further washes with PBS, then resuspended in DMEM/F-12 to a dose of 1 × 10^6^ CFU/mL. To inactive bacteria, the bacterial suspension was heat killed for 60 min at 70 °C after washed with PBS as described. Then it was centrifuged at 4000×*g* for 10 min and resuspended in DMEM/F-12 to have a final inoculum corresponding to 1 × 10^8^ CFU/mL. The doses of active and heat-inactive *E.coli* were selected based on a preliminary study and on studies of Korzekwa et al. [35] and Brauner et al. [25], respectively.

### Experiment design and treatments

To evaluate the impact of cortisol on the inflammatory response of BEEC induced by *E.coli* or LPS, the cells plated on 6-well plates at a density of 1 × 10^6^ cells per well were challenged with 1 × 10^6^ CFU/mL live *E.coli*, 1 × 10^8^ CFU/mL heat-killed *E.coli*, or 1 μg/mL LPS in the control medium or the medium containing cortisol (H0888, Sigma, USA). The concentrations of cortisol from 5 ~ 30 ng/mL were within the physiological levels in cows. The selection of 5, 15, and 30 ng/mL cortisol was based on a previous report of our lab [17]. For detecting the gene expressions of the cytokines, the cells were collected at 2, 4, and 6 h after live *E.coli* challenge or 2, 6, and 18 h after heat-killed *E.coli* or LPS stimulation.

### Cell viability assay

The previous study in our lab demonstrated that 5, 15, and 30 ng/mL cortisol had no effect on BEEC viability [17]. To determine the effect of heat-killed *E.coli* on cell viability, the Cell Counting Kit-8 (CCK-8) obtained from Dojindo Molecular Technologies, Inc. (Kumamoto, Japan) was used. The cells were plated on 96-well plates at a density of 1 × 10^3^ cells per well and grown to 80% confluence in a 37 °C, 5% CO_2_ incubator. Then the cells were treated with 1 × 10^8^ CFU/mL heat-killed *E.coli* for 0, 2, 6, 9, 12, 16, 18, 24, 48, and 60 h, followed by the addition of CCK-8 to each well. After incubation for 2 h at 37 °C, the optical density (OD 450 nm) was detected with a microplate reader (Tecan, Austria).

The effect of live *E.coli* on cell viability was evaluated by trypan blue assay (C0011, Beyotime, China). The cells were incubated in the 6-well plates (1 × 10^6^ cells per well) and grown to 80% confluence. After treatment with 1 × 10^6^ CFU/mL *E.coli* for 6 h, the cell suspension was mixed with trypsin blue according to the manufacturer’s protocol. Cells were counted using a cytometer (TC10, Bio-Rad).

### RNA extraction and quantitative PCR

After treatment, cells were washed with 1 mL of PBS. Total RNA was extracted using the TRIzol reagent (15,596,018, Thermo, USA) according to the manufacturer’s protocol. The extracted RNA was quantified using a Nanodrop 2000 spectrophotometer (Thermo, USA). Total RNA (1 μg) was reverse transcribed into cDNA with the PrimerScript RT regent Kit gDNA Eraser (DRR047A, Takara, Japan). Real-time PCR was carried out by using SYBR Premix Ex Taq™ II (RR820A, TaKaRa, Japan) on the CFX 96 Real-Time PCR Detection System (BIO-RAD, USA) as previously described [34]. The 2^-△△Ct^ method was used to analyze the relative gene expression. The β-actin was used as the internal control. The sequences of the primers were shown in Table 1. The PCR experiments were performed in triplicate.

**Table 1:** The list of primer sequences used for amplification of qPCR

Gene	Primer sequence (5′ → 3′)	Accession number
β-Actin	F: CATCACCATCGGCAATGAGC	NM_173979.3
	R: AGCACCGTGTTGGCGTAGAG	
TLR4	F: GCTCTGCCTTCACTACAGGGACT	NM_174198.6
	R: CTGGGACACCACGACAATAACC	
IL1β	F: TGATGACCCTAAACAGATGAAGAGC	NM _174093.1
	R: CCACGATGACCGACACCACCT	
IL6	F: TGAAAGCAGCAAGGAGACACT	NM_173923.2
	R: TGATTGAACCCAGATTGGAAGC	
TNF-α	F: CCCTTGTTCCTCACCCAC	NM_173966.2
	R: CTCGGCATAGTCCAGGTAG	
IL8	F: TTCCTCAGTAAAGATGCCAATG	NM_173925.2
	R: TGACAACCCTACACCAGACCCA	

### Statistical analysis

Data analysis was performed using IBM SPSS Statistics 21.0 (IBM, NY, USA). All data were expressed as the means ± standard error (SEM). The significance of differences between groups was evaluated by one-way ANOVA, followed by Dunnett’s test. Significance was attributed when a two-sided *p*-value was less than 0.05. Each experiment was repeated three times.

## Data Availability

The datasets used and analysed during the current study available from the corresponding author on reasonable request.

## Abbreviations

BEEC: Bovine endometrial epithelial cells
CCK-8: Cell Counting Kit-8
cDNA: Complementary DNA
DMEM/F-12: Dulbeccos’s Modified Eagle Media/Nutrient Mixture F-12
*E.coli*: *Escherichia coli*
FBS: Fetal bovine serum
LB: Luria-Bertani
LPS: Lipopolysaccharide
qPCR: Quantitative polymerase chain reaction
T3SS: Type III secretion system

## References

[1] Gobikrushanth M, Salehi R, Ambrose DJ, Colazo MG (2016). Categorization of endometritis and its association with ovarian follicular growth and ovulation, reproductive performance, dry matter intake, and milk yield in dairy cattle. Theriogenology.

[2] LeBlanc SJ, Duffield TF, Leslie KE, Bateman KG, Keefe GP, Walton JS, Johnson WH (2002). Defining and diagnosing postpartum clinical endometritis and its impact on reproductive performance in dairy cows. J Dairy Sci.

[3] Sheldon IM, Cronin JG, Healey GD, Gabler C, Heuwieser W, Streyl D, Bromfield JJ, Miyamoto A, Fergani C, Dobson H (2014). Innate immunity and inflammation of the bovine female reproductive tract in health and disease. Reproduction.

[4] Sheldon IM, Price SB, Cronin J, Gilbert RO, Gadsby JE (2009). Mechanisms of infertility associated with clinical and subclinical Endometritis in high producing dairy cattle. Reprod Domest Anim.

[5] Azawi OI (2008). Postpartum uterine infection in cattle. Anim Reprod Sci.

[6] Davies D, Meade KG, Herath S, Eckersall PD, Gonzalez D, White JO, Conlan RS, O'Farrelly C, Sheldon IM (2008). Toll-like receptor and antimicrobial peptide expression in the bovine endometrium. Reprod Biol Endocrinol.

[7] Newton K, Dixit VM (2012). Signaling in innate immunity and inflammation. Cold Spring Harb Perspect Biol.

[8] Moresco EM, LaVine D, Beutler B (2011). Toll-like receptors. Curr Biol.

[9] Herath S, Fischer DP, Werling D, Williams EJ, Lilly ST, Dobson H, Bryant CE, Sheldon IM (2006). Expression and function of toll-like receptor 4 in the endometrial cells of the uterus. Endocrinology.

[10] Cronin JG, Turner ML, Goetze L, Bryant CE, Sheldon IM (2012). Toll-like receptor 4 and MYD88-dependent signaling mechanisms of the innate immune system are essential for the response to lipopolysaccharide by epithelial and stromal cells of the bovine endometrium. Biol Reprod.

[11] Straub RH, Cutolo M (2016). Glucocorticoids and chronic inflammation. Rheumatology (Oxford).

[12] Busada JT, Cidlowski JA (2017). Mechanisms of glucocorticoid action during development. Curr Top Dev Biol.

[13] Barnes PJ (1998). Anti-inflammatory actions of glucocorticoids: molecular mechanisms. Clin Sci (Lond).

[14] Hapgood JP, Avenant C, Moliki JM (2016). Glucocorticoid-independent modulation of GR activity: implications for immunotherapy. Pharmacol Ther.

[15] Stahn C, Lowenberg M, Hommes DW, Buttgereit F (2007). Molecular mechanisms of glucocorticoid action and selective glucocorticoid receptor agonists. Mol Cell Endocrinol.

[16] Kindahl H, Kornmatitsuk B, Gustafsson H (2004). The cow in endocrine focus before and after calving. Reprod Domest Anim.

[17] Dong J, Qu Y, Li J, Cui L, Wang Y, Lin J, Wang H (2018). Cortisol inhibits NF-kappaB and MAPK pathways in LPS activated bovine endometrial epithelial cells. Int Immunopharmacol.

[18] Shen Y, Liu B, Mao W, Gao R, Feng S, Qian Y, Wu J, Zhang S, Gao L, Fu C (2018). PGE2 downregulates LPS-induced inflammatory responses via the TLR4-NF-kappaB signaling pathway in bovine endometrial epithelial cells. Prostaglandins Leukot Essent Fatty Acids.

[19] Chapwanya A, Meade KG, Doherty ML, Callanan JJ, O'Farrelly C (2013). Endometrial epithelial cells are potent producers of tracheal antimicrobial peptide and serum amyloid A3 gene expression in response to E. coli stimulation. Vet Immunol Immunopathol.

[20] Yang W, Zerbe H, Petzl W, Brunner RM, Günther J, Draing C, von Aulock S, Schuberth HJ, Seyfert HM (2008). Bovine TLR2 and TLR4 properly transduce signals from Staphylococcus aureus and E. coli, but S. aureus fails to both activate NF-κB in mammary epithelial cells and to quickly induce TNFα and interleukin-8 (CXCL8) expression in the udder. Mol Immunol.

[21] Lyu A, Chen JJ, Wang HC, Yu XH, Zhang ZC, Gong P, Jiang LS, Liu FH (2017). Punicalagin protects bovine endometrial epithelial cells against lipopolysaccharide-induced inflammatory injury. J Zhejiang Univ Sci B.

[22] Lim KH, Staudt LM (2013). Toll-like receptor signaling. Cold Spring Harb Perspect Biol.

[23] Borish LC, Steinke JW (2003). 2. Cytokines and chemokines. J Allergy Clin Immunol.

[24] Sikora J, Smycz-Kubanska M, Mielczarek-Palacz A, Kondera-Anasz Z (2017). Abnormal peritoneal regulation of chemokine activation-The role of IL-8 in pathogenesis of endometriosis. Am J Reprod Immunol.

[25] Brauner A, Soderhall M, Jacobson SH, Lundahl J, Andersson U, Andersson J (2001). Escherichia coli-induced expression of IL-1 alpha, IL-1 beta, IL-6 and IL-8 in normal human renal tubular epithelial cells. Clin Exp Immunol.

[26] Croxen MA, Finlay BB (2010). Molecular mechanisms of Escherichia coli pathogenicity. Nat Rev Microbiol.

[27] Clements A, Young JC, Constantinou N, Frankel G (2012). Infection strategies of enteric pathogenic Escherichia coli. Gut Microbes.

[28] Pallett MA, Berger CN, Pearson JS, Hartland EL, Frankel G (2014). The type III secretion effector NleF of enteropathogenic Escherichia coli activates NF-kappaB early during infection. Infect Immun.

[29] Kayagaki N, Wong MT, Stowe IB, Ramani SR, Gonzalez LC, Akashi-Takamura S, Miyake K, Zhang J, Lee WP, Muszynski A (2013). Noncanonical inflammasome activation by intracellular LPS independent of TLR4. Science.

[30] Barnes PJ (2010). Mechanisms and resistance in glucocorticoid control of inflammation. J Steroid Biochem Mol Biol.

[31] Chinenov Y, Rogatsky I (2007). Glucocorticoids and the innate immune system: crosstalk with the toll-like receptor signaling network. Mol Cell Endocrinol.

[32] Cruz-Topete D, Cidlowski JA (2015). One hormone, two actions: anti- and pro-inflammatory effects of glucocorticoids. Neuroimmunomodulation.

[33] Sorrells SF, Sapolsky RM (2007). An inflammatory review of glucocorticoid actions in the CNS. Brain Behav Immun.

[34] Cui L, Wang H, Lin J, Wang Y, Dong J, Li J, Li J (2020). Progesterone inhibits inflammatory response in E.coli- or LPS-Stimulated bovine endometrial epithelial cells by NF-κB and MAPK pathways. Dev Comp Immunol.

[35] Korzekwa AJ, Lupicka M, Socha BM, Szczepanska AA, Piotrowicz E, Baranski W (2016). In vitro cow uterine response to Escherichia coli, leukotrienes and cytokines. Vet Immunol Immunopathol.

